# European Culture: Centripetal and Centrifugal Forces

**DOI:** 10.1134/S1019331622100082

**Published:** 2022-09-22

**Authors:** Al. A. Gromyko

**Affiliations:** Institute of Europe, Russian Academy of Sciences, Moscow, Russia

**Keywords:** European culture, humanism, risk society, pop art, modern, postmodern, postmaterialism, avant-garde art

## Abstract

The main features of the transformation of European culture in the modern world are explored. Chronologically, this study is based on the idea of the general humanistic foundations of European culture since the 1980s. The author describes its state during the Cold War, and then analyzes the search for its new meanings, including the concept of the “new Middle Ages” and the theme of fear. Another dimension of the research is the role of the Age of Enlightenment in the history of European culture and its later development in the context of liberalism, consumerism, and individualism. Contemporary European culture is characterized as an overlap of premodern, modern, and postmodern. The problem of Westernization and standardization of culture is dwelt upon together with the shift to different currents of postmaterialism and neo-avant-garde art.

The fate of Europe and European culture is an extremely broad and at the same time specific topic. It is as much historiosophical as it is narrowly specialized, depending on what becomes the object of study: politics or economics, identity or values, fine arts or everyday culture, the spiritual or material sphere, literature or cinema, museums or theater, or the culture of memory or the culture of dialogue. Infinitely much has been written about various aspects of modern European culture, but quite little has been written about this phenomenon comprehensively, for example [*European* …, 2013; Rubinskii, 2002; Rubinskii, 2013].

This article traces a number of leading trends in the transformation of European culture from the era of the Cold War and industrial society to the postbipolar era and the postmodern world using iconic examples. Particular attention is paid to the historical period starting from the 1980s. In the epistemological sense and from the point of view of theoretical approaches to the study of the multifaceted phenomenon of European culture, the author analyzes the ideas proposed in the works of P.Ya. Chaadaev, F.A. Stepun, N.A. Berdyaev, D.S. Likhachev, O. Spengler, A. Toynbee, Z. Freud, J. Ortega y Gasset, U. Eco, E. Husserl, F. Fukuyama, S. Huntington, J. Gray, and others. A great contribution to the study of this phenomenon was made by scientists from the Institute of Europe, Russian Academy of Sciences: N.P. Shmelev, Yu.I. Rubinskii, E.V. Vodop’yanov, and others.

## ***

In recent decades, the Old World has undergone tremendous changes. After the collapse of the Soviet Union in 1991, in addition to Russia, 14 new independent states have appeared on the political map of Eurasia, six of which are within geographic Europe (Estonia, Latvia, Lithuania, Belarus, Moldova, and Ukraine). At the same time, integration processes have been gaining momentum to the west of the Russian borders. Over the past 40 years, the number of countries that are members of the European Union (until 1992, the European Economic Community) has increased from 9 to 28. However, centrifugal forces have not bypassed it, and in 2020 Britain left the EU. Discussions about Europe are inevitably accompanied by long-standing disputes about the boundaries of the Old World, whether geographical, civilizational, political, or value-oriented. In this case, the concept of Greater Europe refers to an extremely heterogeneous civilizational space located between three oceans—the Atlantic, the Arctic, and the Pacific [*Europe*…, 2019].

The adult European 30–40 years ago and such an individual now are people living in different realities. Nevertheless, not so much has changed in the history textbooks over the indicated time, especially if we take the history of Europe until the second half of the 20th century. School textbooks in countries from Lisbon to Vladivostok are essentially products of the same framework cultural matrix, albeit perhaps the most diverse and controversial in the world. Culture is one facet of a “long history,” a history of structures that change extremely slowly. Human behavior and perception of the world have never kept up with the pace of technological development. Moreover, this applies to the identity of each nation and individual person, including the cultural environment in which we are placed from childhood and with which we are permeated. This environment is highly differentiated into “high” and “low” culture, elite and folk, sophisticated and consumerist, local and global.

## EUROPEAN CULTURE IN THE COLD WAR ERA

The words of E. Husserl, stated in 1935, apply to the whole of Greater Europe: no matter how hostile European nations are to each other, they still have an inner kinship of spirit that permeates them all and overcomes national differences [Husserl, 1995, p. 302]. Europeans, heirs of the Greco–Roman and Christian civilizations both before and after 1945, were brought up on classical examples and works of the Renaissance and Enlightenment; literature, poetry, painting, and architecture of the New Age; the “golden” age for Russia of the 19th century and the “Silver Age.” Possibly, the 19th century became the peak of European culture, at least in its “high” component. Then European humanism was almost trampled underfoot by two world wars. The bipolar world has largely politicized European culture but not completely. Almost 70 years after Husserl, the Russian Europeanist Yu.I. Rubinskii stated: “No matter how deep the differences between Europeans, they are related by a common destiny, compatibility, and, moreover, the complementarity of their very rich cultural heritage” [Rubinskii, 2002, p. 60].

Industrial society has become a new “mold” for culture. With the phenomenon of “mass revolt” and accelerated urbanization in the first third of the 20th century, there was a massification of culture, its democratization, emancipation, secularization, and large-scale penetration of “high” culture into the masses. In the second half of the century, Westernization became one of the leading forms of such massification, increasingly slipping into Americanization and standardization, which gave rise to a variety of mass culture—pop culture. There has been a kind of bricolage,^1^ a “rebound,” expressed in the “revenge” of culture when folk culture in its vulgar incarnation crushed “high” culture under itself. As a result, the circle has closed: “high” culture again, as it once was before the “uprising of the masses,” has become the lot of the creative minority, and the minority that stands mainly on national and not on cosmopolitan soil. This parallels the social emancipation of the early 20th century and various revolutions and social cataclysms in the 1980s–2000s.

Culture reflects the course of history and, in many ways, shapes it. Europe of the 1980s was still a postwar phenomenon, part of the world, not only divided by the bipolar era, but also shaped by the great Victory of 1945. However, economically and technologically, Europe was already deeply involved in the process of transition from industrial to postindustrial society and from modernity to postmodernity, including in the area of culture. It was in 1980 that E. Toffler, a classic of futurology, published *The Third Wave* about the postindustrial world [Toffler, 1980]. Nevertheless, the turn of the 1980s–1990s became a real watershed, when the world, after the end of the Cold War and the departure of the Soviet Union into history, began to turn into a global one in terms of trade, market relations, finance, politics, and, of course, culture.

Until the 2010s Europe lived in this global world—the apogee of the neoliberal model of globalization, which by now has largely sunk into oblivion. The history of the last few decades is full of sharp turns. The Western part of the world has parted with the illusions of unipolarity. There was a formation of polycentrism, including its cultural component, for example, such a phenomenon as Indian Bollywood. The global world experienced a shock at the beginning of the 21st century, when the international community was challenged by international terrorism, which destroyed the monuments of ancient and modern culture. Later, most of the planet fell into the “great recession,” and the pandemic that began in 2020 brought the world into a new state of frightening uncertainty.

What is typical of history is that dates and chronological frames are relative. The postindustrial world was formed in the 1970s. D. Bell wrote about this [Bell, 1973], but a decade earlier, a “cultural revolution” (or counterculture revolution) swept through Europe, in some way anticipating the coming changes. It was no less an offspring of the mass consciousness of late industrial society than it was a forerunner of the superliberal individualism that swept the world thereafter. Culture, being in its essence a derivative of the collective and traditional, was increasingly used to please the individual and the private.

Despite the split of the world in the bipolar era, the general humanistic framework of European culture was preserved. Thus, this is evidenced by the cinema art, which is so sensitive to politics: Soviet movies were awarded the American Academy Award four times in different categories; for the last time, in 1981, at the peak of a new round of the Cold War (*Moscow Does Not Believe in Tears* by Vladimir Menshov in the nomination best film in a foreign language). After the resounding success of the Bolshoi Theater tour of London in 1956, theater visits on both sides of the Iron Curtain became fairly commonplace in the following decades.

The Soviet secondary school curriculum, not to mention the faculties of art history, studied the works of Shakespeare and Goethe, as well as other classics of European literature and poetry. In the Soviet Union, the works of A. France and G. de Maupassant, C. Dickens and J. Swift, M. Cervantes and V. Scott, E. Zola and V. Hugo, J. Byron and A. Dumas, O. de Balzac and M. Proust, A. de Saint-Exupery and E.M. Remarque were translated into Russian in large circulations—the list is long. Plays by European playwrights were shown in theaters. According to the memoirs of representatives of the Soviet intelligentsia, they perceived themselves as representatives of European culture, and this was a common occurrence. In general, the interaction of European and Russian cultures has been a two-way process, especially since the time of Peter the Great. European classicism played a huge role in Russian classicism.^2^

In contrast to classical art, fate prepared something else for the Russian avant-garde, primarily abstractionism. The destruction of avant-garde artists by Nikita Khrushchev in the Moscow exhibition complex Manege in 1962, as well as the closing of an exhibition of nonconformist artists in Moscow in 1974 (“bulldozer exhibition”), went down in history. The Russian avant-garde was truly discovered abroad thanks to Sergei Diaghilev’s Russian Seasons in Paris at the beginning of the 20th century, and then in 1979, when a large-scale exhibition “Paris–Moscow” was held in France, at the Pompidou Center. A landmark series of exhibitions of Russian avant-garde art called “The Great Utopia” took place already at the beginning of the post-Soviet period, in 1992, in Frankfurt and Amsterdam, as well as in the United States.

Another example of the state of the cultural space is music, and not necessarily classical. In 1978, with the permission of the Soviet Ministry of Culture, the Boney M. group came to the Soviet Union, and the following year, Elton John gave concerts in Leningrad and Moscow. An even greater public outcry was caused by the performances in the Soviet Union of the rock group Scorpions in 1988. Shortly after that, they would release their new album, which included the iconic song *Wind of Change*. In post-perestroika Russia, tours by contemporary Western musicians would become regular, including Paul McCartney’s concert on Red Square in 2003.

The end of the Cold War at the turn of the 1980s–1990s somewhat smoothed out the contradictions within the Old World, including due to the deideologization of culture. One of the symbols of this was the return of the writer Alexander Solzhenitsyn in 1994 to the new Russia, 20 years after the expulsion of the writer from the Soviet Union. It is appropriate to recall that Solzhenitsyn, the 1970 Nobel laureate in literature, lived in Zurich for several years before moving to the United States and traveled around Western Europe. At first, in the West, the writer was praised for his anticommunist views, but then the opinion about him changed. Long-standing disputes in Russia between the Westerners, the Slavophiles, and the Pochvenniks intertwined in the figure of Solzhenitsyn. In the West, he found shelter, sharply condemned the Soviet system, but at the same time criticized American reality, and began to be perceived as a supporter of “religious–patriarchal romanticism.” Later, Solzhenitsyn talked about a renewed union of the three Slavic republics—Russia, Belarus, Ukraine—and Kazakhstan.^3^ Another iconic figure among those who were expelled from the USSR for anti-Soviet views was the philosopher Alexander Zinoviev. From 1978 to 1999 he lived in Munich. In many ways, following Solzhenitsyn, the trajectory of his views developed from Westernism to Slavophilism.

After the dismantling of the “Iron Curtain,” new cultural faults, already of a different level, could not be avoided over time. Europe, confirming the diagnosis of its eternal internal contradictions, became the site of new dividing lines, and Western Europeans engaged in new social and cultural engineering. The EEC, and then the European Union, created a narrative of a new Europe, the borders of which were equated with the borders of an integration project centered in Brussels. The civilizational boundaries of the Old World were historically mobile: they either narrowed or expanded, but in general, over time, they absorbed more and more new lands. However, never until the 1990s any attempt was made to designate the borders of Europe with the outer contour of a postmodern regional integration association instead of the civilizational, historical, political, social, and cultural space of the former European metropolises, in other words, at first speculatively sharply narrow the European space to the territory of the EU and then expand “Europe” on the basis of rules constructed and formally legalized in the EU. In Russia, however, the long-standing historiosophical dispute resumed, in which Russia and the West were opposed.

It is true that culture has permeated humanity throughout its existence, as well as the fact that every century, every era has its own culture. In this, continuity did not conflict with diversity and renewal. No matter how one interprets the European culture of Modern and Contemporary times, no matter how one arranges it according to national regiments and epochs, it was generally accepted that it had a common denominator—the value of Christianity and humanism. Of course, European culture has more than once encountered its antipodes, including Nazism and fascism, which almost destroyed it. While claiming their own ethics and aesthetics, they were not manifestations of a “different culture”; it was an anticulture directed against humanity.

Various forms of massification of culture in the industrial and postindustrial eras did not always lead to its degradation. For example, the replication of the *Dove of Peace* by Pablo Picasso did not deprive this work of a humanistic charge. At the same time, there has always been a danger of emasculating the value of one image or another as a result of its repeated and inappropriate reproduction. So, countless T-shirts with the image of Mona Lisa or Ernesto Che Guevara, sold around the world, led to the vulgarization of these images rather than to the popularization of symbols of beauty and passionarity. After the collapse of the Soviet Union, there was a kind of mutation of Soviet symbols: within the framework of Sots Art, its interpenetration with Western pop art took place. Lenin was found side by side with the inscription Coca-Cola; Stalin, with Marilyn Monroe; portraits of Lenin and Stalin were placed against the backdrop of the Marlboro cigarette brand; Gorbachev was depicted in the style of Warhol’s *Marilyn Diptych*, and the sculptural composition *Worker and Collective Farm Woman* was crowned with the head of Mickey Mouse.

## SEARCH FOR NEW MEANINGS

The modern era of universal and all-pervading information, standardization, and unification has called into question the possibility of mass education of a cultured person in a European way. The conditions for this were difficult: the originality of the 1980s in the history of the Old World was followed by a period of illusions and then disappointments—including those of epic proportions, like the Great Recession or a pandemic. Many load-bearing structures of modern European culture began to be comprehended precisely in the 1980s. It is no coincidence that the Frenchman J. Baudrillard published his famous work *Simulacres et simulation* [Baudrillard, 1981] in 1981. One of its theses has become a reference: “We live in a world where there is more and more information and less and less meaning.”

Over the past decades, attempts have been made in the western part of the Old World to give European culture new meanings. One of them was the concept of the “new Middle Ages,” which was developed and popularized by U. Eco, including the work “The Middle Ages Have Already Begun” (1993) [Eco, 1994]. In it, he argued with an earlier dystopia by R. Vacca *The Near Medieval Future* [Vacca, 1971], in which the author predicted the retreat of the modern technological era into a gloomy past.^4^ Eco himself was more optimistic and viewed modernity as a “continuous period of transition,” when, as in the Middle Ages, the task was not to preserve the past, but to bring the conflict between the old and the new under control and create an adaptation mechanism. These arguments of Eco are consonant with the work of other thinkers devoted to various aspects of risk. So, in 1986, the canonical book by W. Beck *Risk Society: Towards Another Modernity* [Beck, 1986] was published. E. Giddens studied the phenomenon of risk in his works on late modernity [Giddens 2000].

The past few decades have related the feelings of a European with medieval themes of fear, even with the expectations of the end of the world, at least the end of the world to which they are accustomed. Such feelings were bizarrely intertwined with periods of euphoria. However, a new spiritual upsurge always ended in a return of pessimism. In the 1980s Europe was afraid of a third world war between the Soviet Union and the United States because of the deployment of nuclear missiles on its territory and on both sides of the Iron Curtain. In 1986, the man-made disaster of Chernobyl broke out. The euphoria of the end of the Cold War was replaced by a cold shower of conflicts in the post-Soviet space, the Yugoslav wars, and the struggle to preserve the territorial integrity of Russia itself. Perestroika illusions were overshadowed by the dramas and tragedies of millions of people who found themselves on the wrong side of the borders after the collapse of the Soviet Union.

At the turn of the millennia, expectations of a happy “end of history” were replaced by gloomy forecasts of a “clash of civilizations.” Approximation in the chronology of the magic number “2000” was associated by some with the Last Judgment and by others with the “computer Apocalypse.” No sooner had the new millennium begun than the problem of international terrorism rose from Russia to a new level after 9/11. The project of the so-called world caliphate of ISIS^5^ was directed to the destruction of European culture and its physical extermination. In 2008–2009 Europe was rocked by the Great Recession, and in 2020, by the COVID-19 pandemic.

Assessing the current state of Europe, other intellectuals did not start from the Middle Ages but from another period in its history—the Enlightenment, which laid the foundation for the political philosophy and ideology of liberalism, culture, and then the cult of personality and freedom of the individual. J. S. Mill and I. Berlin, separated by a century, spoke about the problems that accumulated in this area. Promoting liberal ideas, each in his own way, they recognized the need for collectivity in culture, belonging to one community. The problem of recent decades in the life of the Old World is largely connected with the emasculation of the principles of classical liberalism, with the vulgarization and absurdization of ideas about freedom, with the transformation of liberalism into a secular religion, and with the exhaustion of the universality of the Enlightenment project. J. Gray called this kind of thinking hyperliberalism, which produced cultural deconstruction and freed the individual from cultural identities [Grey, 2018; Grey, 1999; Gray, 1993; Bellamy, 1992].^6^

Manifestations of such hyperliberalism began to multiply, for example, the requirement introduced in several European countries to remove symbols of faith from public places and from the outer vestments of a person. Thus, from the point of view of conservative social thought, and even common sense, Europe deprived itself of cultural roots and cultural immunity and became vulnerable to the expansion of other cultures, including the fundamentalist part of Islamic culture. The system of values of the modern European has increasingly represented a deformed and unbalanced set of ideas, dominated not by liberalism in its classical form but by neoliberalism to the detriment of the conservative and collectivist traditions of social thought and consciousness [Gromyko, 2020].

Since the 1990s European culture and European identity has been tested by unprecedented migration processes. After the collapse of the Soviet Union, several tens of millions of former Soviet citizens ended up in new states in which they became a national minority. This especially affected more than 20 million Russians. At the same time, paradoxically, the new Russia, whose borders were pushed to the east, ethnically turned into a more European state than the Soviet Union, as the proportion of Russians, whose worldview was based on European culture, increased dramatically in the country (up to 80%).

In Western and Central Europe, an unprecedented migration crisis unfolded later. It peaked in 2015, when several million people from the Middle East and Africa arrived in the EU as a result of “uncontrolled migration.” Germany was at the center of these events. Disputes based on different ideas about state sovereignty and the relationship between the interests of “indigenous people” and “outsiders” became a bone of contention in relations between EU member states. The Mediterranean Sea, whose basin was the cradle of several ancient civilizations, including the Greco–Roman, became the grave for tens of thousands of refugees who dreamed of finding the promised land in Europe. Since then, large-scale migration problems have not stopped, inevitably exacerbating the issue of European self-consciousness, culture, and identity.

Criticism of the ideas of the Enlightenment, a by-product of which in the 20th century became Nietzsche’s superman and mass consumer society, sounded in many modern literary works, for example, in P. Süskind’s *Perfume* [Süskind, 1985]. The hero of his novel Grenouille, who killed himself, is a kind of superman in reverse. The theme of smell in this novel is, in fact, an instrument of mass consumption driven to hysteria. W. Golding’s book *Lord of the Flies*, which later became a cult classic, appeared in 1954, but the writer received the Nobel Prize for his work in the critical 1980s [Golding, 1954]. Many years later, in 2009, *The Times* would list this work as one of the best 60 books of the previous 60 years.^7^ Its meaning lies not in the praise of Man—this is not a book about Prometheus or Icarus—but about the fall of man.

The category of empire has become another direction in understanding the modern culture and identity of Europe. Literature appeared devoted to the European Union as an empire, including elements of culture and identity, for example [Zielonka, 2006; Tevdoi-Bulmuli, 2019]. Here it is appropriate to mention the phenomenon of “enlargement fatigue” in the European Union. It outlined the limits of the EU as an empire ennobled and “neomedieval.”

## “BREAD AND CIRCUSES!”

Modern European culture appears as an interweaving and stratification of the old and the new: premodern, modern, and postmodern. From the depths of history, the attitude “Bread and Circuses!” was transferred to Europe of the Newest Time, which took on an exaggerated mass-consumer character. One of its personifications was malls—huge shopping and entertainment centers that brought the cultural industry to the absolute, to the merging of mass culture with the entertainment industry, including cinema, mostly American. The share of Hollywood movies on the screens of Western Europe increased in 1975–1995 from 41 to 75% (by ticket sales). It was a one-way street: even Britain’s share of audio-music exports to the United States in 1986–2001 decreased from 30 to 1% [*European*…, 2013, p. 262]. The European film industry was caught between the Hollywood model and the “art house.”

The thinkers of the Frankfurt School (T. Adorno, M. Horkheimer, H. Marcuse, and others) argued about the pitfalls of massification and standardization of culture back in the distant 1920s. The great folk culture, which hundreds of years ago gave rise to a comical, amusing, and carnival culture in Europe, has almost degenerated in the era of postmodernity and “numbers.” After the collapse of the socialist camp, the cultural industry swept over the post-Soviet space. Throughout Europe, theater has given way to the onslaught of cinema and other forms of visual entertainment. Cosmopolitanism led to massification in architecture as well. N. Foster’s buildings turned into a symbol of prestige, but had nothing to do with national identity.

In recent decades, the religious component of culture and self-consciousness in Europe has increasingly moved to second and third roles, except for Russia and a number of other countries. However, the belief in the other world, characteristic of religious thinking, was somewhat replaced by other phenomena, for example, the virtual reality of computer games, and the feeling of a believer’s involvement in one flock was replaced by a feeling of the interconnection of social network users, where you can, as in confession, pour out your soul without seeing and without even knowing the interlocutor. Christian humanism, with the fading of the religiosity of European society, gave way to humanism “universal”; there was a unification of values in the spirit of the “end of history,” which is somewhat akin to the expectations of the end of the world characteristic of religious thinking. As people used to go to church en masse, then they also sat en masse in front of television pop art, which is another powerful tool for moral and aesthetic degradation.

This problem has long been pointed out by those who played the role of guardians of “high” European culture. Among them is K. Popper, who in 1994 gave a detailed interview to *Reset* magazine. He talks about the devastating impact of the “blue screen” on children and adolescents [Popper, 2007]. One of the embodiments of the negative side of the Americanization of European culture was the MTV channel (youth music and reality shows), created in 1981. In other countries, numerous offspring from its have appeared, for example, MTV-Russia, which opened in 1998. They were distinguished by the dominance of base products, about which Popper warned.

In the late-perestroika Soviet Union and post-Soviet Russia, a wave of occultism, mysticism, and magic swept over the television screen and concert halls. Millions of educated people fell under the influence of “healers” and “psychics,” such as A. Kashpirovskii and A. Chumak. Pseudoscience flourished. It turned out to be a clear illustration of the theory of cultural development by J. Frazer, the author of the famous work *The Golden Bough*, who built his research according to the formula “magic–religion–science” [Frazer, 1894]. The postmodern wave of massification of culture in the form of pop culture has become a relapse, a rollback of culture in its development. There was a movement back from science to religion and then to magic. It should be noted that in moments of social crises, irrational value systems, including religious ones, more than once have played the role of a social “airbag.” However, if a society “gets stuck” in this rollback, then there is a danger of falling into the archaic. Therefore, it is quite understandable that modern European culture pays so much attention to the concepts of archaization and barbarization, including the vulgarization of the Russian language, and “political barbarism” (international terrorism), and “ecological barbarism,” etc.

Classics of postmodern European culture turned to these and similar motifs. The meaning of J. Barnes’s novel *A History of the World in 10½ Chapters* [Barnes, 1989] was the story of a man’s journey to paradise, which turned out to be a consumerist and unbearable place. The English writer turned out to be more perspicacious than F. Fukuyama, who in the same year published the essay “The End of History?,” which marked the beginning of a well-known discourse [Fukuyama, 1989]. In 1988, i.e., five years before the publication of S. Huntington’s article “The Clash of Civilizations?” [Huntington 1993], *Satanic Verses* by S. Rushdie was published—a work built on the image of the conflict of cultures and civilizations [Salman, 1988].

The year 1989 in the history of Europe is forever associated with the fall of the Berlin Wall as a symbol of the division of the world in the bipolar era. This and the accompanying events sparked an upsurge of speculation about what would come “after.” For example, in Germany, the phenomenon of “postwar literature” arose, one of the personifications of which was the novel by G. Grass *The Wide Field* (in Russia, the novel is better known under the name *Long Conversation*, proposed by the translator B.N. Khlebnikov) [Grass, 1995]. The end of the Cold War led to a clash in the European culture of passionarity and idealism with everyday life, prosaic reality, and the imperfection of human nature. The successive cycles of expectations and disappointments in recent decades are obvious. Examples are the late Soviet Union and the new Russia of the 1990s, Germany after unification and still divided German society thirty years later, perestroika illusions in the spirit of “Europe is our common home” and the resumption of confrontation between Russia and the West, the idealism of the “Arab Spring” and the tragedies of entire peoples of North Africa and the Middle East that replaced it, and anticipation of the “European dream” of the leading role of the European Union in the 21st century and the subsequent series of dangerous crises.

At the same time, the improvement of technology has continued, and technological progress has left less and less time for realizing reality. As a result, the theme of confrontation between man and machine has regained popularity. In the cinema, it is vividly embodied in blockbusters about merciless terminator robots. COVID-19 also brought with it a new kind of Luddism—the “rebellion of people against machines”: in 2020 in Europe, due to fear of a pandemic, modern Luddites destroyed 5G mobile network towers. This is also reflected in the shift from the culture of consumption to postmaterialism taking place in the European mass consciousness, as evidenced by the ideology of European environmentalists and the “greens.”

A very crucial topic is the culture of memory. It is almost inevitable that with the passage of time, new generations increasingly regard the events of the receding past as something abstract. The oblivion of wars, on the one hand, served to reconcile the once warring states, as happened with France and Germany; on the other hand, it deprived people of “immunity” against the revival of militaristic sentiments. Such militarization is well traced in the history of the EU in recent years and decades. Russia, perhaps, today is the only country in Europe in which, on a systematic basis, work continues to preserve the memory of the Second World War (the Great Patriotic War) as a “living history.”

It cannot be said that modern European art does not pay attention to antiwar themes. However, often this is outrageous, grotesque, and conscious provocation aimed at emotional shake-up, the purpose of which is not so much a reminder of the fragility of the world as drawing attention to the newfangled representatives of art. One example is the exhibition “Jake and Dinos Chapman: ‘The End of Fun’” in St. Petersburg, which took place in 2012 in the Hermitage. The composition of the installations of many figurines, a kind of bestiary in which the Nazis kill each other, was designed by the authors to depict hell on earth. The Chapmans’ creative work is based on allusions referring to the series of engravings by F. Goya “Caprichos” and “Disasters of War,” as well as to the work of I. Bosch. At the same time, the scandalous presentation of such exhibitions leads, as a rule, to hypertrophy of form at the expense of content and meaning.

## ***

Europe and European culture over the past few decades have been deeply immersed in the reality of postmodernism; its new offshoots have emerged—postpostmodernism, transhumanism, and posthumanism. The humanistic foundations of the European civilization of Modern and Contemporary times, rooted in antiquity and Christianity, today coexist with modern mass culture and “digital” society with all their light and dark sides. The cultural resistance to stress of European peoples largely depends on the national literary, theatrical, and cinematographic schools. The leading national museums remain the bastion of high art, of which Russia can be proud. In our country, there has been a rapid increase in the attendance of museums and theaters in recent years. Thus, in 2018, 140 million visitors to art exhibitions were registered, and the audience of theaters amounted to 40 million spectators.^8^

In the cultural space, an active creative search continued, often far from unambiguous. There was a boom in private theaters in Russia: in Moscow and St. Petersburg, there were about 60 of them by the end of the 2010s. The same applies to private museums of contemporary art, among which the Garage Museum in Moscow’s Gorky Park has become one of the most famous. The V-A-C Contemporary Art Foundation has created a multiformat contemporary art space (art center) in the building of the former HPP-2 opposite the Kremlin. Since 2005, the Moscow Biennale of Contemporary Art has been held, within the framework of which much attention was paid to Sots Art as the most famous postmodern direction of Soviet art abroad in the 1970s–1980s. Russia has not bypassed Manifesta, a pan-European biennale that was first held in 1996 in Rotterdam. In 2014, St. Petersburg became the first Russian city to host Manifesta 10.

The topic of a common cultural space, based on European humanism, was constantly raised. The exhibition “Facing the Future: Art of Europe 1945–1968,” which was held in 2017 at the Moscow Pushkin State Museum of Fine Arts, became a unique project. The foreign partners of the project were the BOZAR Fine Arts Center (Brussels) and the ZKM Arts and Media Center (Karlsruhe). The project focused on the postwar art and culture of 18 countries of Western and Eastern Europe (neo-avant-garde art), dedicated to the themes of antiwar and youth rebellion, the horrors of violence, and new searches in the realm of realism and idealism. The pandemic in 2020–2021 hit hard on exhibition and museum projects. The large-scale exhibition “Diversity, Unity, Modern Art of Europe: Moscow, Berlin, Paris” after a long forced pause, nevertheless opened at the Tret’yakov Gallery in November 2021.

Whether the European cultural space will continue to experience fragmentation, politicization, and, to a large extent, degradation is an open question. Can classical culture continue to serve as its “cementing mortar”? Is it possible to harmonize national traditions with a “digital” world full of conflicts? It seems that the colossal cultural heritage of Europe still has a margin of safety to withstand bad taste, primitivization, clip thinking, and deconstruction of high and popular culture.

## Footnotes

1 From the French *bricolage,* a billiard term that means to send the ball to the board, so that, having rebounded from it, it hits the pocket.

2 From the author’s conversations with Yu.A. Borko, an outstanding Soviet and Russian Europeanist, professor, and chief researcher at the RAS Institute of Europe.

3 A. I. Solzhenitsyn, “How can we equip Russia?,” Komsomol’skaya Pravda, Sep. 9 (1990). http://www.solzhenitsyn.ru/proizvedeniya/publizistika/stati_i_rechi/v_izgnanii/kak_nam_obustroit_ rossiyu.pdf?ysclid=l37jbz6mj7

4 Long before the European intellectuals of recent decades, for example, Nikolai Berdyaev wrote about the “new Middle Ages” in his work “The New Middle Ages” (1924). Berdyaev compared his time with the period of late antiquity.

5 This organization is banned in Russia.

6 Grey J. “The problem of hyper-liberalism,” TLS, Mar. 30 (2018). https://www.the-tls.co.uk/articles/john-gray-hyper-liberalism-liberty/. See also [Grey, 1999; Grey, 1993; Bellamy, 1992].

7 “The best 60 books of the past 60 years,” Sunday Times, Aug. 3 (2009).

8 “Attendance at Russian theaters in 2018 broke Soviet records,” Interfax Jan. 16 (2019). https://www.interfax-russia.ru/kaleidoscope/poseshchaemost-rossiyskih-teatrov-v-2018-godu-pobila-sovetskie-rekordy
